# A rapid action plan to improve diagnosis and management of lipodystrophy syndromes

**DOI:** 10.3389/fendo.2024.1383318

**Published:** 2024-06-04

**Authors:** Lindsay T. Fourman, Josivan Gomes Lima, Vinaya Simha, Marco Cappa, Saif Alyaarubi, Renan Montenegro, Baris Akinci, Ferruccio Santini

**Affiliations:** ^1^ Metabolism Unit, Massachusetts General Hospital and Harvard Medical School, Boston, MA, United States; ^2^ Hospital Universitário Onofre Lopes, Departamento de Clinica Medica, Universidade Federal do Rio Grande do Norte, Natal, Brazil; ^3^ Division of Endocrinology, Mayo Clinic, Rochester, MN, United States; ^4^ Research Area for Innovative Therapies in Endocrinopathies Bambino Gesù Children’s Hospital, IRCCS, Rome, Italy; ^5^ Pediatric Endocrinology, Oman Medical Specialty Board, Muscat, Oman; ^6^ Brazilian Group for the Study of Inherited and Acquired Lipodystrophies (BRAZLIPO), Clinical Research Unit, Walter Cantidio University Hospital, Federal University of Ceará/Ebserh, Fortaleza, Brazil; ^7^ Dokuz Eylul University Health Campus Technopark (DEPARK), Dokuz Eylul University, Izmir, Türkiye; ^8^ Department of Research Programs, Technological Research, Izmir Biomedicine and Genome Center (IBG), Izmir, Türkiye; ^9^ Obesity and Lipodystrophy Center, Endocrinology Unit, University Hospital of Pisa, Pisa, Italy

**Keywords:** delay in diagnosis, disease management, lipodystrophy, screening, clinical assessment

## Abstract

**Introduction:**

Lipodystrophy syndromes are rare diseases that can present with a broad range of symptoms. Delays in diagnosis are common, which in turn, may predispose to the development of severe metabolic complications and end-organ damage. Many patients with lipodystrophy syndromes are only diagnosed after significant metabolic abnormalities arise. Prompt action by clinical teams may improve disease outcomes in lipodystrophy syndromes. The aim of the Rapid Action Plan is to serve as a set of recommendations from experts that can support clinicians with limited experience in lipodystrophy syndromes.

**Methods:**

The Rapid Action Plan was developed using insights gathered through a series of advisory meetings with clinical experts in lipodystrophy syndromes. A skeleton template was used to facilitate interviews. A consensus document was developed, reviewed, and approved by all experts.

**Results:**

Lipodystrophy is a clinical diagnosis. The Rapid Action Plan discusses tools that can help diagnose lipodystrophy syndromes. The roles of clinical and family history, physical exam, patient and family member photos, routine blood tests, leptin levels, skinfold measurements, imaging studies, and genetic testing are explored. Additional topics such as communicating the diagnosis to the patients/families and patient referrals are covered. A set of recommendations regarding screening and monitoring for metabolic diseases and end-organ abnormalities is presented. Finally, the treatment of lipodystrophy syndromes is reviewed.

**Discussion:**

The Rapid Action Plan may assist clinical teams with the prompt diagnosis and holistic work-up and management of patients with lipodystrophy syndromes, which may improve outcomes for patients with this rare disease.

## 1 Introduction

Lipodystrophies are a group of heterogeneous, rare, and irreversible conditions characterized by an absence of subcutaneous fat (1, 2). The pattern of fat loss can either be across the whole body [generalized lipodystrophy (GL)] or in specific areas [partial lipodystrophy (PL)]. Both forms of lipodystrophy syndromes (GL or PL) may be genetic or acquired, leading to four major categories: congenital generalized lipodystrophy (CGL), familial partial lipodystrophy (FPLD), acquired generalized lipodystrophy (AGL), and acquired partial lipodystrophy (APL) (3, 4). Typical characteristics of these four major categories are summarized in **Table 1** (5–13). In addition to these classical categories, additional etiologies such as progeroid syndromes, complex genetic syndromes, autoimmune syndromes, and lipodystrophy induced by myeloablative therapy can also manifest with GL or PL.

**Table 1:** Clinical features of four major subtypes of lipodystrophy syndromes.

Clinical feature	Generalized lipodystrophy (GL)		Partial lipodystrophy (PL)	
	Congenital generalized lipodystrophy (CGL)	Acquired generalized lipodystrophy (AGL)	Familial partial lipodystrophy (FPLD)	Acquired partial lipodystrophy (APL)
Fat loss and distribution pattern	Near total absence of body fat starting at birth or shortly after	Progressive loss of fat leading to near-complete lack of adipose tissue	Partial loss of fat predominantly affecting the limbs	Gradual loss of adipose tissue from head downwards
Family history	Yes, consanguinity (usually AR)	No	Yes (usually AD)	No
Associated diseases and comorbidities	Metabolic abnormalities are severe and usually start early in childhood Subtype specific features can be distinctive*	Low serum complement 3 and 4 (C3 and C4); perilipin 1 autoantibodies Can be associated with autoimmune diseases^#^ Metabolic abnormalities are usually severe	Metabolic abnormalities are common Metabolic abnormalities may start later than CGL but can be as severe as CGL in adulthood	Low serum complement 3 (C3) levels, glomerulonephritis Metabolic abnormalities are less common, but can vary in severity

AD, autosomal dominant; AR, autosomal recessive. *i.e., patients with CGL4 present with myopathy, skeletal abnormalities, pyloric stenosis, gastrointestinal motility problems, and cardiac arrhythmias. ^#^e.g., juvenile dermatomyositis, autoimmune hepatitis, type 1 diabetes, panniculitis. In addition to these classical categories, there is additional etiology including progeroid syndromes, complex genetic syndromes, autoinflammatory syndromes, and myeloablative therapy induced lipodystrophy that can present with GL and PL.

Patients with lipodystrophy syndromes present with a broad range of symptoms. Clinical characteristics of lipodystrophy syndromes are heterogeneous and may depend on molecular etiology in genetic cases (14). Adipose tissue loss leads to ectopic fat accumulation which, in turn, triggers the development of severe insulin resistance and metabolic disease (15). Reduced leptin secretion contributes to the pathogenesis of lipodystrophy by adversely affecting appetite control, glucose and lipid homeostasis and metabolism (16, 17). In later stages of the disease, symptoms can include severe metabolic abnormalities (e.g., severe insulin resistance and difficult-to-treat diabetes, severe hypertriglyceridemia) and end-organ complications (e.g., non-alcoholic steatohepatitis (NASH), nephropathy, pancreatitis, cardiovascular disorders, neuromuscular system abnormalities) (1, 14, 15, 18–22).

While an international multi-society guideline exists (4) that covers the diagnosis and management of lipodystrophy syndromes, there is still a great amount of variation in the care that patients with lipodystrophy syndromes receive, including the speed with which they receive it. Despite a gradually increasing awareness of lipodystrophy syndromes among clinicians, it is still common for patients to be diagnosed only after they develop severe metabolic abnormalities and organ complications. Delays in diagnosis expose patients to the risk of developing severe metabolic disease and end-organ damage, which have already developed at the time of diagnosis in many cases (19, 23, 24). To address this knowledge gap, we aimed to create a Rapid Action Plan to support clinicians with limited experience in lipodystrophy syndromes by providing expertise from leading clinical teams. The goal of this plan is to reduce the time it takes for patients with lipodystrophy syndromes to receive a comprehensive diagnosis followed by the care and holistic support that they need.

## 2 Methods

This Rapid Action Plan was developed using insights gathered through a series of advisory meetings with clinical experts in lipodystrophy syndromes. A consensus meeting was held to initiate development of the Rapid Action Plan document at which time a group of international experts in lipodystrophy syndromes (United States, Brazil, Italy, Turkey, and Oman) was invited to discuss the key priorities for clinicians in the first 100 days after seeing a patient with clinical suspicion for the diagnosis. The rationale of the project, its scope and the role of expert contributors were further presented at this meeting. There was broad agreement from experts across this field that additional tools to help clinicians understand key indicators, priority tests and patient follow-up would be valuable. All parties have reviewed and endorsed the steps within this plan. The steps outlined reflect a consensus from experts on what action they would recommend given their experience. It is designed to be a reference tool for clinical teams that can complement national and international guidelines.

A skeleton template was used to facilitate interviews with experts. The first two sections of the skeleton were developed to review general information on lipodystrophy syndromes such as explaining the main subtypes of lipodystrophy. The next section was about the Rapid Action Plan focusing on diagnosing and supporting a patient with a suspicion of lipodystrophy syndromes. The aim of this section was to generate an action plan for clinical teams that can help reach a diagnosis without delay. This section was developed to answer specific questions such as:

- What physical findings and clinical features should raise suspicion for lipodystrophy syndromes?
- What are the other differential diagnoses that are likely to be considered by peers and experts? What is the list of conditions that need to be included in differential diagnosis?
- How can I confirm the diagnosis and screen for complications and comorbidities?
- How can I educate patients about the diagnosis and available support?

Experts were asked to provide information on tools that can support prompt diagnosis and referral. The role of physical signs and clinical history, skinfolds and radiology, laboratory testing, leptin level, and genetic testing was explored. Treatment strategies for lipodystrophy syndromes following metabolic risk stratification were also discussed.

After all interviews were completed, experts were asked for a second round of meetings to review outputs from initial interviews. Based on feedback from experts, revisions were made. A consensus document was developed, reviewed, and approved by all experts.

## 3 Results

### 3.1 Diagnosing lipodystrophy syndromes

#### 3.1.1 Physical and metabolic signs

In patients with GL, the condition may be relatively apparent due to absence of fat in the whole body, prominent veins, and increased muscular appearance. However, parents and patients can easily get accustomed to this physical appearance if GL is not diagnosed at birth, and this may delay diagnosis until the development of potentially severe metabolic abnormalities. Children with GL more frequently present with elevated liver enzymes and severe hypertriglyceridemia compared to their counterparts with PL. Abdominal distension and protrusion of the umbilical scar are common and may help with diagnosis.

PL may be more challenging to identify as fat loss is selective. Phenotype also varies according to sex, with men usually presenting with a less prominent fat distribution abnormality and less severe metabolic profile than women. In certain subtypes of lipodystrophy syndromes, such as FPLD type 2 (Dunnigan variety), abnormal fat accumulation, particularly in the face and neck, can be remarkable. Patients with FPLD may exhibit significant muscularity and phlebomegaly in the limbs, which can be remarkable in lower extremities. In pediatric patients, metabolic abnormalities can be absent so adipose tissue distribution should be carefully considered.

Other associated signs and symptoms include insatiable appetite, skin findings (e.g., acanthosis nigricans, hirsutism in women, eruptive xanthomata), skeletal abnormalities (e.g., bone cysts, scoliosis), other organ abnormalities based on molecular etiology (in genetic cases), and autoimmune features (in acquired cases).

There are several disorders that need to be considered in the differential diagnosis of GL and PL. A list of common entities is listed in **Table 2** (11, 25).

**Table 2:** Differential diagnosis of GL and PL.

Generalized lipodystrophy (GL)	Partial lipodystrophy (PL)
• Conditions associated with very lean body shape (e.g., malnutrition/starvation, anorexia nervosa, cachexia caused by various etiology such as cancers, inflammatory disorders, hyperthyroidism, adrenal insufficiency, HIV associated wasting, diencephalic cachexia, etc.) • Constitutional thinness • Severe insulin resistance due to insulin receptor mutations • Acromegaly/pseudoacromegaly	• Truncal obesity • Metabolic syndrome • Type 2 diabetes (especially when poorly controlled and/or associated with hepatic steatosis and high triglycerides) • HIV-associated lipodystrophy • Lipodystrophy-like phenotype • Cushing syndrome

- Lack of subcutaneous fat is the most critical manifestation.
- Any person with partial or complete lack of subcutaneous fat should be evaluated for the diagnosis of lipodystrophy syndromes.
- Be wary of features of metabolic syndrome in the absence of increased adiposity or high BMI.
- Lipodystrophy syndromes should be considered when metabolic symptoms are disproportionate to body size, including diabetes with high insulin requirements, hypertriglyceridemia, fatty liver disease, or polycystic ovary syndrome (PCOS).

#### 3.1.2 Clinical and family history

Taking a detailed clinical and family history of the patient can help in diagnosing lipodystrophy syndromes and understanding the type of lipodystrophy a patient may have, especially for genetic forms of the disease. The family history should include questions about body shape as well as a history of known (or suspected) metabolic comorbidities or other clinical characteristics. It is important to remember phenotypic differences between men and women as it can be easy to overlook a male relative with lipodystrophy syndromes. Although no other family member with lipodystrophy can be detected in acquired lipodystrophies, it is important to assess for a history of autoimmunity and radiation and rule out HIV infection.

- Collating photos of patients through their lifetime can be very helpful. Sensitively ask for photos of the patient before/after symptoms (and ideally in different states of dress).
- The absence of adipose tissue can be identified at birth or within the first year of life in CGL, while fat loss develops at any time in life in AGL. In FPLD, fat loss can be detected in childhood, but it typically becomes prominent around puberty. Fat loss can start at any time in life in APL (usually in childhood, adolescence, or young adulthood).
- Photos of family members can also be helpful to assess and determine if a suspected lipodystrophy syndrome is genetic.
- Personal or family history of autoimmune diseases can be helpful.

#### 3.1.3 Routine tests

In general, upon initial evaluation of the patient (and at follow-up appointments), routine laboratory tests should be considered to assess diabetes [e.g., fasting glucose levels, glycated hemoglobin (HbA1c)] and lipid abnormalities [fasting serum lipids (especially triglyceride levels)]. Oral glucose tolerance test (OGTT) can be considered on a case-by-case basis. In addition, liver function tests along with hepatic ultrasound and/or liver elastography to identify signs of non-alcoholic fatty liver disease (NAFLD) or hepatic fibrosis are recommended to evaluate hepatic disease. A baseline electrocardiogram (ECG) is recommended. As part of cardiac examination, an echocardiogram can be helpful to detect heart abnormalities (e.g., signs of cardiomyopathy).

- Laboratory tests should be considered to assess for insulin resistance, diabetes, and lipid abnormalities (high triglycerides and low HDL cholesterol are typical findings).
- Liver function tests along with hepatic ultrasound and/or elastography can identify signs of NAFLD and hepatic fibrosis.

#### 3.1.4 Leptin assay

While leptin deficiency is a hallmark in the pathology of lipodystrophy syndromes, consensus among the group was that a given leptin level cannot be used to rule in or out a lipodystrophy diagnosis.

- Low leptin level is supportive but not diagnostic of lipodystrophy syndromes.
- Normal or high leptin levels do not rule out a lipodystrophy diagnosis.

#### 3.1.5 Skinfold measurement

The panel thought that it is important to include objective measures of body composition as part of screening tools to aid in the diagnosis of lipodystrophy syndromes. This is particularly the case in PL where fat loss can be subtle. The panel agreed that skinfold measurement can be useful (particularly alongside other parameters) in the diagnosis of lipodystrophy syndromes, However, it is considered a less precise option when compared to alternative methods for measuring body fat. Where the technology and resources allow, alternatives should be considered before skinfold measurement including dual-energy x-ray absorptiometry (DXA) (see below).

- Mid-thigh skinfold thickness is easy to obtain and can be useful, but it is a rather imprecise option compared with alternatives for measuring body fat. Skin fold thickness values of the anterior thigh <10mm in adult men and <22mm in adult women are supportive information for the diagnosis of GL and FPLD (3).

#### 3.1.6 Radiology

Where available, imaging modalities should be seen as an important objective tool to support the diagnosis of lipodystrophy syndromes. While magnetic resonance imaging (MRI) can be considered a useful tool, the panel noted that it is far less practical and more costly than dual energy X-ray absorptiometry (DXA). If physicians consider it impractical and burdensome to use MRI, it is suggested that this be reserved for research or exceptional circumstances, and that DXA is a suitable, cost-effective and appropriate alternative.

- Where available, imaging modalities should be seen as an important objective tool to support diagnosis of lipodystrophy syndromes.
- DXA is a suitable, cost-effective and appropriate strategy to assess fat quantity and distribution.
- Although clear diagnostic definitions are not established, a very low percentage of body fat may suggest GL, and a Fat Mass Ratio (the ratio between percent of the trunk fat mass and the percent of the lower-limb fat mass) higher than 1.2 in females may suggest FPLD (26).
- MRI is a useful tool to assess body composition but can be less practical and more costly.

#### 3.1.7 Genetic testing

Lipodystrophy in a younger patient or with a positive family history (or consanguinity) may suggest a genetic etiology. Genetic testing in patients with suspected congenital or familial lipodystrophy syndromes can be used to confirm the diagnosis. However, a negative genetic test does not rule out an inherited form of the disease since some genes involved in the pathogenesis of lipodystrophy syndromes have yet to be identified. Furthermore, some forms of genetic lipodystrophy syndromes are polygenic in origin with affected genes not typically included in standard lipodystrophy panels. In patients suspected of lipodystrophy syndromes in whom genetic testing is negative, acquired forms of lipodystrophy syndromes should additionally be considered.

- Preference of the format of genetic testing continues to vary across localities.
- Among the expert panel reviewing this resource, diagnostic genetic panels were generally preferred over single gene testing.
- As caveats to genetic testing, some genes involved in the pathology of lipodystrophy syndromes are yet to be identified and some forms of genetic lipodystrophy syndromes can be polygenic.
- Whole Exome Sequencing (WES) was typically reserved for patients highly suspected of inherited lipodystrophy syndromes for whom the genetic panel did not yield results.
- As technology continues to improve, WES may become increasingly a viable option for routine use.

**Figure 1:**
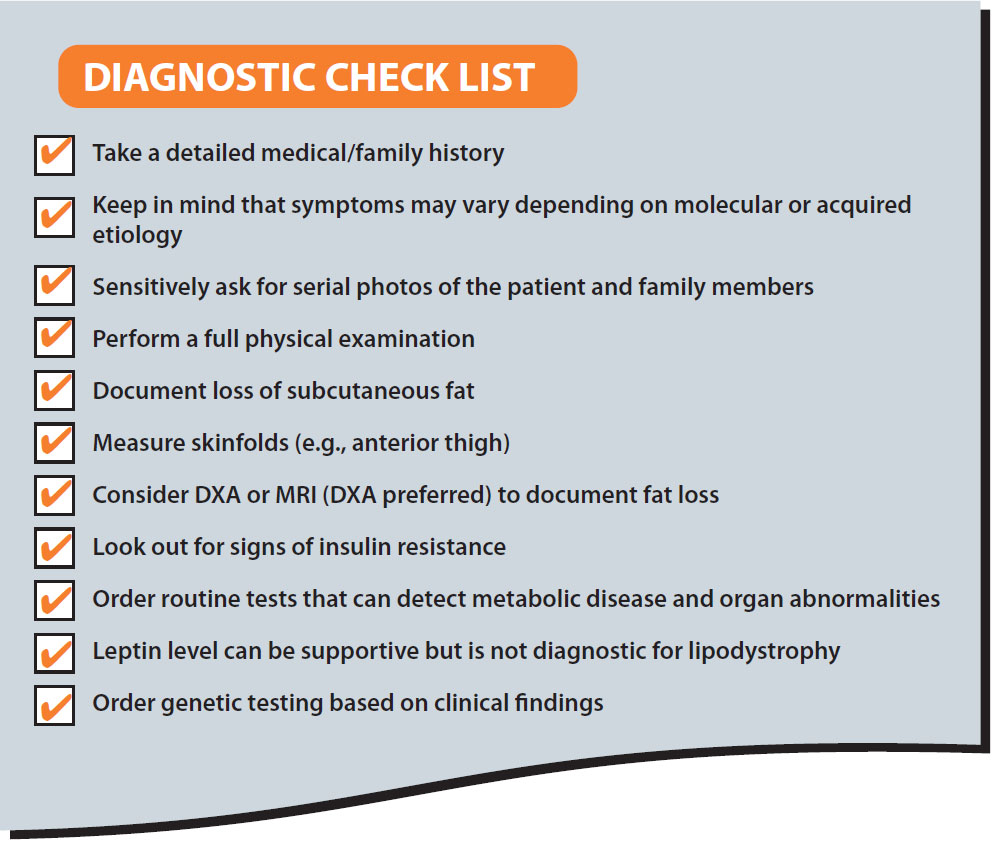
Diagnostic check list.

A diagnostic checklist is presented in **Figure 1**.

### 3.2 Communicating the diagnosis to the patient and families

A diagnosis of lipodystrophy is a life-changing moment and careful consideration must be given as to how best to communicate this to patients and their family members. The following are useful areas to consider on this topic:

• Several discussions may be needed.

Diagnosis is often a relief for people with lipodystrophy syndromes; however, there is often a lot of information to completely understand and it is likely to take several discussions/consultations before the patient is comfortable with the meaning of the diagnosis.

• Include other members of the multidisciplinary team.

Delivering the diagnosis alongside other specialist(s) from the multidisciplinary team that will likely be involved in the management of the patient can be helpful in some cases.

• Discuss proactive management.

The diagnosis can be framed as an opportunity to be proactive with respect to monitoring and managing risk factors for potential comorbidities.

• Distress over irreversible changes to appearance is common.

It is important that the person with lipodystrophy syndromes understands that it is generally not possible to regain lost fat. This can be very distressing and psychological support should be offered. There are also cosmetic options such as reconstruction surgery and fillers that could be considered.

• Consider genetic counselling in relevant cases.

The diagnosis of a heritable form of lipodystrophy syndromes is accompanied by specific considerations, and genetic counselling should be offered.

### 3.3 Referrals for patients

Referral to an expert center, where there is a multidisciplinary team with expertise in treating lipodystrophy syndromes, is highly recommended by the expert panel. If a nearby expert center does not exist, efforts should be made to consult with specialists who have experience in treating patients with lipodystrophy syndromes.

• Referral to an expert center is highly recommended.

### 3.4 Screening and monitoring for metabolic dysfunction and organ abnormalities

#### 3.4.1 Insulin resistance, diabetes mellitus, and associated complications

Once a diagnosis of lipodystrophy syndrome is made, it is important to assess patients for insulin resistance, diabetes mellitus, and associated complications. For most patients, following international/national diabetes treatment guidelines is recommended (4, 27, 28). Even if initial laboratory results appear normal, regular monitoring should be conducted.

- Check fasting glucose (consider ordering fasting insulin alongside) as well as HbA1c at least annually.
- Screen patients for complications of insulin resistance and diabetes.

#### 3.4.2 Lipids and pancreatitis

Dyslipidemia (high triglycerides, low HDL-cholesterol) is a major comorbidity of lipodystrophy syndromes which can lead to development of eruptive xanthomas and episodes of acute pancreatitis and is associated with increased cardiovascular risk (20). Following international/national treatment guidelines for hyperlipidemia is generally recommended (4, 29).

- Monitor fasting lipid panel at least annually and repeat with occurrence of abdominal pain or xanthomata.

#### 3.4.3 Liver disease

Liver disease (severe liver steatosis leading to NASH and eventually liver cirrhosis) remains a major cause of mortality in patients with lipodystrophy syndromes and should be carefully monitored (30).

- Monitor liver enzymes at least annually.
- Consider elastography, liver ultrasound, MRI (with Dixon fat) or magnetic resonance elastography at diagnosis, and then every few years or sooner as clinically indicated.
- Liver biopsy is performed as clinically indicated.

#### 3.4.4 Cardiovascular disease

Several forms of lipodystrophy syndromes are associated with heart disease (e.g., cardiomyopathy, arrhythmias, conduction abnormalities, coronary artery disease) due to frequent and severe metabolic complications and underlying molecular etiology (21, 22, 31–36).

- Blood pressure should be measured at every visit.
- Baseline ECG is recommended and should be repeated as clinically indicated.
- Echocardiogram should be considered as clinically indicated.
- Symptoms of coronary artery disease should be assessed carefully, and further testing should be considered based on clinical findings.
- Consider Holter ECG or ECG with exercise test to evaluate for arrhythmias in select patients.
- Risk of sudden death syndrome should not be overlooked in lipodystrophy syndromes.

#### 3.4.5 Kidney disease

Chronic kidney disease (CKD) is a major cause of mortality in lipodystrophy syndromes. CKD is frequent and has an early onset in patients with lipodystrophy syndromes, and thus vigilance is recommended (37).

- Urine protein/creatinine ratio and serum creatinine levels should be assessed at the time of diagnosis and repeated at least annually.

#### 3.4.6 Reproductive system

Reproductive dysfunction such as PCOS, oligo/amenorrhea, reduced fertility, and hirsutism is commonly detected in women with lipodystrophy syndromes. Also, women with lipodystrophy syndromes are theoretically at increased risk for preeclampsia, miscarriage and macrosomia due to poor metabolic control. Early adrenarche, true precocious puberty, or central hypogonadism also may occur in children with GL (1, 15, 38–40).

- Gonadal steroids, gonadotropins, and pelvic ultrasonography can be helpful in identifying reproductive dysfunction.
- Pubertal staging should be performed annually in children.

#### 3.4.7 Hunger and other additional symptoms

Patients with lipodystrophy syndromes, especially GL, are typically hyperphagic due to leptin deficiency (41–45). However, monitoring hunger can be a challenge. While there was no overall consensus on how best to measure hyperphagia, the panel noted that it may be helpful to consider the use of questionnaires such as the Three-Factor Eating Questionnaire (46).

Comorbidities and other impairments of lipodystrophy syndromes that may affect patient quality of life include, but are not limited to, social anxiety and limitations with symptoms or physical appearance leading to inability to work and a reluctance or inability to socialize, chronic pain that can affect a patient’s ability to carry out basic tasks, and fatigue (47, 48).

- Patients with lipodystrophy syndromes, especially GL, typically have hyperphagia due to leptin deficiency which can have implications for patients, family members and caregivers.
- Additional symptoms of lipodystrophy may include anxiety, depression, chronic pain, and fatigue.

### 3.5 Treating lipodystrophy syndromes

#### 3.5.1 Diet

Generally, patients are advised to follow diets with balanced macronutrient composition (3, 4). Energy-restricted diets improve metabolic abnormalities and may be appropriate in adults. Very-low-fat diets should be used in patients with severe hypertriglyceridemia (triglycerides > 1000 mg/dL). In the absence of severe hypertriglyceridemia, patients should replace refined carbohydrates with unsaturated fat and protein. Alcohol intake should be limited. A dietician should be consulted for specialized dietary needs, especially in infants and young children (3, 4, 49). Overfeeding should be avoided. Medium-chain triglyceride oil formulas can provide energy without raising triglycerides in infants.

- Most patients are recommended to follow a calorically restricted diet with balanced macronutrient composition; however, it may be difficult to control hunger in a leptin deficient state.
- Referral to a dietician is strongly recommended.
- Alcohol and smoking should be avoided.

#### 3.5.2 Exercise

Patients with lipodystrophy syndromes should be encouraged to exercise in the absence of specific contraindications. Patients with subtypes of lipodystrophy syndromes predisposed to cardiomyopathy and/or arrhythmias should undergo cardiac evaluation before initiating an exercise regimen. Contact sports should be avoided in patients with severe hepatosplenomegaly and in patients with CGL who have lytic bone lesions. Referral to an exercise specialist may be appropriate.

- Exercise is encouraged in the absence of specific contraindications.

#### 3.5.3 Role of metreleptin

**Figure 2:**
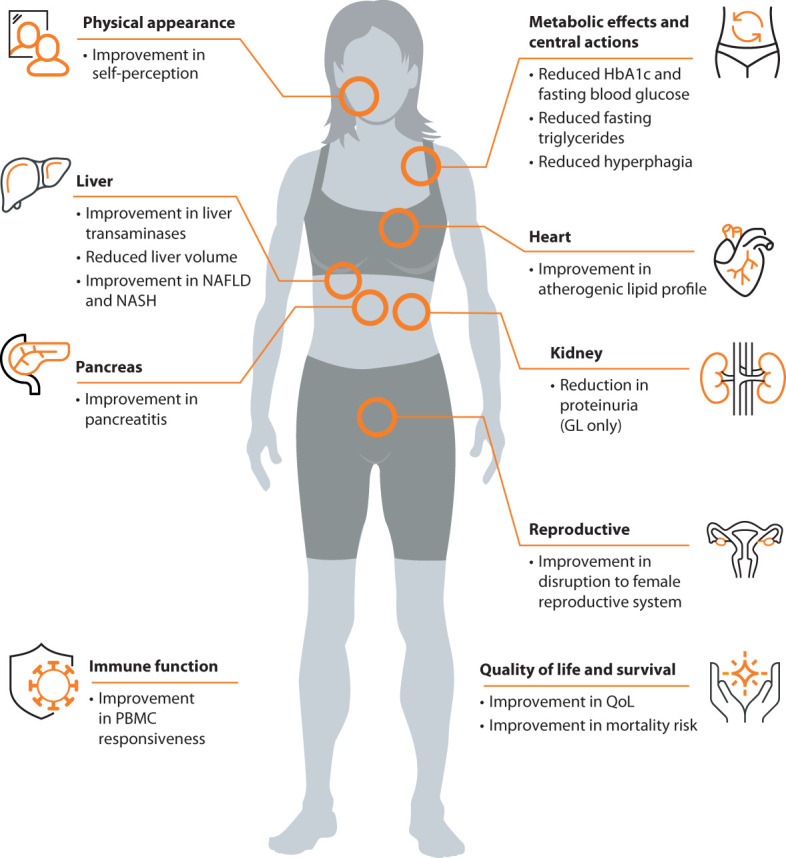
Clinical effects of metreleptin in patients with lipodystrophy syndromes.

Metreleptin, a recombinant analog of the human hormone leptin, is an orphan drug used to treat complications of leptin deficiency in lipodystrophy syndromes. In the US, metreleptin is approved as an adjunct to diet as replacement therapy to treat the complications of leptin deficiency in patients with CGL or AGL (50). In Europe and Brazil, metreleptin is indicated as an adjunct to diet as a replacement therapy to treat the complications of leptin deficiency in adults and children 2 years of age and above with confirmed CGL or AGL (51, 52). Metreleptin is also approved for this indication in adults and children 12 years of age and above with confirmed FPLD or APL in whom standard treatments have failed to achieve adequate metabolic control (51). The effects of metreleptin treatment are summarized in **Figure 2**.

- The expert panel concluded that, where available, metreleptin should be used in accordance with local guidelines.

#### 3.5.4 Management of diabetes with standard approaches

In addition to the 2016 multi-society lipodystrophy guideline, international/national diabetes treatment guidelines can be helpful (4, 53). Metformin is the most commonly used agent to improve insulin resistance and glycemic control (3, 4). Thiazolidinediones may improve metabolic complications in PL (4, 54–56) but should be used with caution in GL. While animal data suggest partial reversal of metabolic disturbances in GL mouse models with thiazolidinediones (57), clinical experience in patients with GL is very limited (54, 58, 59). Preliminary data from case reports (60) and a case series indicate sodium-glucose cotransporter 2 (SGLT2) inhibitors can help improve glycemic control among patients with PL (61). Due to severe insulin resistance, many patients with lipodystrophy syndromes will need large amounts of insulin to control their blood glucose. Concentrated insulins should be considered (4, 62). In patients with PL, reduction of body mass index (BMI) may reduce the risk of metabolic comorbidities (63). Glucagon-like peptide-1 (GLP-1) receptor analogues may improve metabolic levels (64, 65). It is not known whether GLP-1 receptor analogues affect fat distribution in lipodystrophy syndromes. Some experts felt that GLP-1 receptor agonists may help reduce localized fat accumulation (e.g., on the abdominal fat) and may also help manage hyperphagia in some cases, although these effects have not been systematically studied in patients with lipodystrophy syndromes. Prior episodes of acute pancreatitis and severely elevated triglyceride levels can be limiting factors to GLP-1 receptor agonist use (66, 67).

- Treat diabetes per guidelines. Maintain oversight but include referral to an endocrinologist as part of management.

#### 3.5.5 Management of lipid abnormalities with standard approaches

Lifestyle modifications are essential in the management of hypertriglyceridemia (see Diet and Exercise sections above). Statins should be used concomitantly with lifestyle interventions in the setting of hypertriglyceridemia, high low-density lipoprotein cholesterol (LDL-C), or diabetes mellitus for cardiovascular risk reduction. Fibrates are commonly used to treat severely elevated triglycerides though physicians should confer with their local prescribing guidelines regarding use in children. Long-chain omega-3 fatty acid use also may be helpful in treating hypertriglyceridemia. Although no studies are available in patients with lipodystrophies, a previous large-scale clinical study (68) reported cardiovascular benefits in patients with fasting triglyceride levels of 135–499 mg/dL following treatment with icosapent ethyl. However, the use of these agents may be limited in patients with lipodystrophies who are at higher risk of developing arrhythmias, as an increased risk of atrial fibrillation has been reported after the use of omega-3 fatty acids, particularly in high doses (69–71).

- Treat dyslipidemia aggressively using lifestyle modification and statins with or without fibrates.
- Cardiovascular disease risk calculators may not necessarily be accurate in patients with lipodystrophy syndromes.

#### 3.5.6 Management of liver disease with standard approaches

The panel agreed that monitoring for liver disease is critical in patients with lipodystrophy. NAFLD may develop early and progress rapidly to NASH and cirrhosis in this population. Weight loss, exercise, and avoidance of excess alcohol are mainstays of treatment per international/national guidelines (3, 4, 72). However, from a patient perspective, significant weight loss can be an undesired outcome in lipodystrophy syndromes, especially in patients with GL.

- Monitor and treat for liver disease per guidelines.
- Consider referral to a hepatologist as part of management.

#### 3.5.7 Management of cardiovascular disease with standard approaches

The panel considers patients with lipodystrophy syndromes to be at high risk of cardiovascular disease and believes that an aggressive risk mitigation and management strategy is needed. In addition to the 2016 multi-society lipodystrophy guidelines (4), the American Diabetes Association (ADA) guideline can be helpful for cardiovascular risk management and hypertension treatment among patients with diabetes (27, 29). Patients with subtypes of lipodystrophy syndromes predisposed to cardiomyopathy or arrhythmia should undergo more detailed cardiac evaluation (32, 33). This is especially important before initiating an exercise regimen.

- Monitor and treat for cardiovascular disease per guidelines.
- Consider referral to a cardiologist as part of management.
- Strongly advise against smoking and assist with smoking cessation.

#### 3.5.8 Management of kidney disease with standard approaches

CKD has a complex background in lipodystrophy syndromes and can be severe and rapidly progressing (37). Depending on the subtype, the etiology of CKD may vary. Although patients with lipodystrophy syndromes may develop CKD in the absence of diabetes, consensus from the group was to treat many of them in a way similar to patients with CKD caused by diabetes (unless CKD is caused by a specific form of kidney disease such as C3-positive membranoproliferative glomerulonephritis) according to international/local guidelines (despite lack of evidence in patients with lipodystrophy syndromes) (73, 74). Angiotensin-converting enzyme inhibitors (ACEi) or angiotensin receptor blockers (ARB) in addition to sodium-glucose cotransporter 2 (SGLT2) inhibitors can be considered if urine protein is elevated (73).

- Monitor and treat for kidney disease per guidelines.
- Consider referral to nephrologist if patient is hypertensive, has elevated creatinine or low estimate glomerular filtration rate (eGFR), or proteinuria.

#### 3.5.9 Management of reproductive health with standard approaches

In patients with low leptin levels, irregular menses may normalize on leptin replacement therapy (40). In patients who are not candidates for or whose menses do not normalize on leptin replacement, if a patient is amenorrhoeic due to a low leptin level and does not improve after leptin treatment, or cannot access leptin treatment, consider hormone replacement therapy– an estrogen patch (less harmful than oral estrogens on triglycerides) and oral progesterone, according to international/local guidelines (4, 75). PCOS in lipodystrophy syndromes is generally managed in line with international guidelines (76). Oral estrogens are generally contraindicated in the presence of severe hypertriglyceridemia (4). Patient attempting pregnancy should be referred to a reproductive clinic.

- Maintain oversight but consider referral to a reproductive endocrinologist or gynecologist as indicated.

## 4 Discussion

As a rare disease, lipodystrophy syndromes often pose a diagnostic and treatment challenge. In this regard, an international chart review study (19) involving five treatment centers in Brazil, Turkey and the United States has illustrated that patients commonly experience delays of several years before receiving a definitive diagnosis of lipodystrophy syndrome. Although fat loss is known to be prominent at birth in CGL and around puberty in FPLD, first symptoms of lipodystrophy were typically identified during childhood (mean age: 9.2 years) among patients with GL and during early adulthood (mean age: 24.7 years) among those with PL. More importantly, from the time symptoms were first noted, it took an average of 3.1 years in GL and 9.0 years in PL for physicians to diagnose them accurately. Delays in diagnosis and intervention predispose patients with lipodystrophy syndromes to irreversible end-organ damage, and as such comprehensive, multi-disciplinary diagnostic and treatment plans are critically needed. We developed the Rapid Action Plan to enable clinical teams to promptly diagnose and holistically manage patients with lipodystrophy syndromes. This set of guidance from experts is intended to enhance outcomes for individuals afflicted by this disease.

It is important to recognize that lipodystrophy syndromes are heterogeneous, resulting in a wide spectrum of clinical presentations. Nonetheless, regional or generalized absence of subcutaneous fat is the sine qua non of lipodystrophy and should be assessed for on physical exam. Clinical history is also important to differentiate this diagnosis from other possibilities, and to distinguish genetic versus acquired etiologies. Basic laboratory testing and imaging are useful adjuncts to the history and physical exam and can help to risk stratify patients for cardiometabolic complications. Insulin resistance, diabetes mellitus, hypertriglyceridemia, ectopic fat accumulation (e.g., hepatic steatosis), and low leptin level are common characteristics of the disease. However, these features may not be present in patients with all forms of lipodystrophy syndromes and are not specific for the condition.

Several tools can help to verify clinical suspicion for lack of subcutaneous fat. Skinfold measurement is an important part of objective assessment that is portable, fast, and affordable. Specifically, mid-thigh skinfolds with a cut-off of less than 10 mm in adult men and less than 22 mm in adult women (corresponding to approximately the 10^th^ percentile of the US population) are useful to support the diagnosis (3). However, skinfold thicknesses are highly operator-dependent, do not give a comprehensive representation of overall fat distribution, and may be prone to error in patients with low body fat. Thus, imaging modalities are preferred, where available, as discussed below.

Among the most important and practical imaging modality noted by this expert panel was the DXA scan (77–79). Where skinfold measurement provides a simple on-the-spot measurement for body fat, DXA allows for the quantification of not only body fat, but also lean mass over time. Also, fat shadow images, which are color-coded representations highlighting only the fat tissue, can be generated from DXA images to facilitate visualization of fat distribution (80). Body composition can alternatively be assessed by computed tomography (CT) and MRI scans. Among the panel, use of MRI was generally recommended to obtain objective ratios of different fat compartments in patients with for PL (81, 82) and to provide information regarding residual mechanical fat in patients with CGL (83, 84). MRI also can determine the lipid content of tissues, solid organs (such as the liver) (85), and bone marrow fat (84) using the Dixon method or spectroscopy.

Despite the need to be mindful of a ‘false negative’, genetic testing remains an important supportive diagnostic and prognostic tool. A genetic test where the commonly known variants (e.g., *LMNA*) are negative should not rule out familial or congenital forms of lipodystrophy syndromes, which remain clinical diagnoses. On the other hand, positive genetic testing can be confirmatory of the diagnosis and should prompt a clinician to screen for complications directly or indirectly associated with molecular etiology and to offer genetic testing to affected family members.

Given the progressive nature of lipodystrophy syndromes, regular screening for metabolic abnormalities and end-organ complications is imperative. The comprehensive follow-up algorithm encompasses various components, including detection of insulin resistance, diabetes, complications associated with diabetes, lipid abnormalities, pancreatitis, liver disease, cardiovascular disease, kidney disease, reproductive system abnormalities, and other comorbidities driven by molecular or acquired etiologies. Emerging evidence emphasizes the importance of cardiovascular health in patients with lipodystrophy syndromes. Patients with lipodystrophy syndromes are at risk of coronary artery disease as a result of severe insulin resistance and poor metabolic control (22, 86). It should be noted that patients with *LMNA* mutations are more likely to develop cardiac disease than others with FPLD (33). Moreover, certain types of lipodystrophy syndromes (e.g., CGL4) are linked to potentially fatal cardiac rhythm changes and prompt diagnosis is therefore vital to minimize risk of sudden cardiac death (31, 32). It is critical to identify and to treat these arrhythmias before they lead to sudden cardiac death.

The current management strategy for lipodystrophy syndromes primarily focuses on metabolic health. Maintaining a controlled diet is key for individuals with lipodystrophy syndromes, although this can be challenging for some patients given a lack of fat and insatiable appetite due to leptin deficiency. Standard medications to treat metabolic disease (e.g., diabetes, hyperlipidemia) can be used with limited efficacy (56, 61, 64, 87). Recombinant leptin, metreleptin, is used as an adjunct to diet as a replacement therapy to treat metabolic complications of lipodystrophy syndromes. The clinical development program for metreleptin includes a pivotal study integrating data from two trials (NIH 991265/20010769; Clinical Trials IDs: NCT00005905 and NCT00025883) and a supportive study (FHA101; Clinical Trials ID: NCT00677313). In patients with GL, the NIH 991265/20010769 study showed a mean absolute reduction of 2.2% in HbA1c levels and a mean relative reduction of 32.1% in plasma triglycerides (88). The supportive study FHA101 yielded consistent efficacy results, albeit with a smaller number of patients (89). Discontinuation of insulin, oral antidiabetic medications, and lipid-lowering therapies was observed in a significant percentage of GL patients after starting metreleptin treatment (88). In patients with PL, metreleptin treatment resulted in significant reductions in HbA1c, fasting triglycerides, and liver volume. A subgroup of patients with baseline HbA1c ≥ 6.5% or plasma triglycerides ≥ 500 mg/dL experienced an absolute reduction of 0.9% in HbA1c and a relative reduction of 37.4% in triglycerides (90). Further studies at the NIH and other treatment centers showed that metreleptin decreased hyperphagia (17), improved insulin sensitivity (91, 92), reduced liver steatosis and improved NASH score on biopsy specimens (30, 93). Improvements in reproductive abnormalities in women (40) and reduction in proteinuria in patients with GL (94, 95) were also observed. While limited, real-life studies have supported the robust effectiveness of metreleptin in GL, whereas the impact of treatment has been heterogeneous in patients with PL. Araujo-Vilar et al. (96) reported a decrease from 11.8% to 6.7% in average HbA1c and a 78% reduction in triglycerides in patients with CGL. In a real-world experience analysis of 53 patients (28 GL and 25 PL) from four countries (France, Spain, Italy, and the UK) (97), one year of metreleptin treatment resulted in a mean reduction of 53% in triglycerides and a 1.9% point decrease in HbA1c in subjects with GL. In patients with PL, the mean percentage reduction in triglycerides was 22% and mean decrease in HbA1c was a 0.5%. A recent multicenter retrospective observational cohort study of 47 patients with lipodystrophy (28 GL and 19 PL) who started metreleptin therapy in France between 2009 and 2020 (98) reported significant improvements in HbA1c (from 8.4% to 6.8%) and fasting triglycerides (from 3.6 mmol/L to 2.2 mmol/L) after one year of treatment with metreleptin, with sustained efficacy thereafter. Additionally, a significant decrease was noted in liver enzymes. However, the impact of treatment was heterogeneous in patients with PL, and overall changes in HbA1c within this subgroup were not significant. Among patients with PL, 61% were responders regarding glucose homeostasis, and 61% were responders regarding hypertriglyceridemia at year 1, with those with more severe metabolic disease and lower leptin at baseline, as well as those with preserved β-cell functions, likely being better responders. Metreleptin has a black box warning for the risk of anti-metreleptin antibodies with neutralizing activity and risk of lymphoma in the US and is available only through a restricted program (50). Metreleptin is subject to additional monitoring in Europe (51). Other potential adverse reactions include hypersensitivity reactions, acute pancreatitis associated with discontinuation of metreleptin, hypoglycemia with concomitant use of insulin and other anti-diabetics, T-cell lymphoma, immunogenicity, and serious and severe infections. Also, data from clinical trials do not support safety and efficacy in patients with HIV-related lipodystrophy. A summary of the reported clinical effects of metreleptin in patients with lipodystrophy syndromes are shown in **Figure 2**.

Lipodystrophy syndromes not only impacts physical health but can also significantly affect mental well-being and quality of life (47, 48). Therefore, it is essential to assess patients’ psychological health and, where available, patients should be referred to a psychologist or counselor for support. Given that many patients may experience body dysmorphia, referral to a plastic surgeon or aesthetic specialist may be appropriate to address these concerns.

In a rare disease setting, consultation with dedicated specialized centers hold significant potential to reduce or even prevent lengthy diagnostic and treatment journeys. There are several ways to find the nearest specialist or specialist center for lipodystrophy syndromes. Because lipodystrophy specialists can be found via publications, running a PubMed search may be helpful. In Europe, there are specialist centers listed on the European Lipodystrophy Consortium’s (ECLip) website (https://www.eclip-web.org/lipodystrophies/). Also, European Reference Networks (ERN) for rare diseases might be helpful; the endocrinology ERN has a main thematic group on ‘genetic disorder of glucose & insulin homeostasis’ (MTG3) (https://endo-ern.eu/rare-genetic-disorders-of-glucose-insulin-homeostasis/). In the United States (US), the Endocrine Society website hosts a member-exclusive community to connect with specialists (DocMatter tool) (https://www.endocrine.org/membership/endoforum). In the Middle East, there is growing collaboration around lipodystrophy [Arab Society for Pediatric Endocrinology and Diabetes (ASPED)] (https://asped.net/). In Brazil, BrazLipo (https://brazlipo.org/) exists as a reference point for professionals and the public.

In conclusion, the complexity of lipodystrophy syndromes poses a challenge to clinicians with limited expertise in the field. The Rapid Action Plan may prove helpful for clinical teams seeking to promptly diagnose and holistically manage patients with lipodystrophy syndromes. This information has the potential to enhance outcomes for individuals with this rare disease.

## Data availability statement

The original contributions presented in the study are included in the article/supplementary material, further inquiries can be directed to the corresponding author/s.

## Author contributions

LF: Writing – original draft, Writing – review & editing. JL: Writing – original draft, Writing – review & editing. VS: Writing – original draft, Writing – review & editing. MC: Writing – original draft, Writing – review & editing. SA: Writing – original draft, Writing – review & editing. RM: Writing – original draft, Writing – review & editing. BA: Writing – original draft, Writing – review & editing. FS: Writing – original draft, Writing – review & editing.
